# Physicians’ Understanding and Practices of Pharmacovigilance: Qualitative Experience from a Lower Middle-Income Country

**DOI:** 10.3390/ijerph17072209

**Published:** 2020-03-25

**Authors:** Rabia Hussain, Mohamed Azmi Hassali, Anees ur Rehman, Jaya Muneswarao, Furqan Hashmi

**Affiliations:** 1 Department of Social and Administrative Pharmacy, School of Pharmaceutical Sciences, Universiti Sains Malaysia, Penang 11800, Malaysia; azmihassali@gmail.com (M.A.H.); aneesurrehmanr90@gmail.com (A.u.R.); rahulraoraorao@gmail.com (J.M.); 2 University College of Pharmacy, University of the Punjab, Lahore 54000, Pakistan; furqan.pharmacy@pu.edu.pk

**Keywords:** physicians, Pakistan, pharmacovigilance, qualitative interview, DRAP

## Abstract

Developed countries have established pharmacovigilance systems to monitor the safety of medicines. However, in the developing world, drug monitoring and reporting are facing enormous challenges. The current study was designed to explore the challenges related to the understanding and practices of physicians in reporting adverse drug reactions in Lahore, Pakistan. Through the purposive sampling technique, 13 physicians were interviewed. All interviews were audio-recorded, transcribed verbatim, and analyzed for a thematic content analysis. The thematic content analysis yielded six major themes: (1) Familiarity with medication safety and adverse drug reaction (ADR) concept, (2) Knowledge about pharmacovigilance activities, (3) Practices related to ADR reporting, (4) Barriers impeding ADR reporting, (5) Acknowledgement of the pharmacist’s role, and (6) System change needs. The majority of the physicians were unaware of the ADR reporting system; however, they were ready to accept practice changes if provided with the required skills and training. A lack of knowledge, time, and interest, a fear of legal liability, poor training, inadequate physicians’ and other healthcare professionals’ communication, and most importantly lack of a proper reporting system were reported as barriers. The findings based on emerging themes can be used to establish an effective pharmacovigilance system in Pakistan. Overall, physicians reported a positive attitude towards practice changes, provided the concerned authorities support and take interest in this poorly acknowledged but most needed component of the healthcare system.

## 1. Introduction

An adverse drug reaction (ADR) is defined by World Health Organization (WHO) “as a response to a drug that is noxious and un-intended and occurs at doses normally used in man for prophylaxis, diagnosis or therapy of disease, or for modification of physiological function” [1]. An adverse drug reaction (ADR) is one which has an unknown etiology, causing an enormous fiscal burden on both the society and healthcare system and contributes to around 5–20% of hospitalizations worldwide [1,2,3,4].

During the post-marketing phase of an approved drug, spontaneous adverse drug reaction reporting (SADR) is used for the risk-benefit evaluation and monitoring of new drugs [5]. The field of pharmacovigilance (PV) deals with reports that raise concerns over the post-marketing safety issues of drugs that result in an adverse drug reaction. The World Health Organization (WHO) defines PV as “the science and the activities related to the assessment, detection, understanding and the prevention of the harmful results or any adverse drug-related issues”; hence, the ADR reports constitute the core data sources for pharmacovigilance systems [1]. Many developed countries have successfully established strong pharmacovigilance systems [6] meant to report suspected ADRs that are encountered by healthcare professionals in their clinical practice [7].

Among all healthcare providers, physicians (Phy) are considered as the frontline healthcare providers who can detect and report an ADR, as their bedside evaluation of the patient plays a crucial role in such detection [8]. Therefore, the involvement of physicians is crucial regarding adverse drug reaction reporting [9]. It is a fact that the success of any pharmacovigilance system lies in the participation of all healthcare experts, especially pharmacists and physicians who are considered the best experts to take care of the activities relevant to pharmacovigilance. However, many studies have revealed that insufficient knowledge and awareness about ADR reporting and the attitude of the physician is the main factor in the under-reporting of ADRs [10,11,12,13]. 

Like the majority of the pharmacovigilance systems in lower and middle-income countries, where a lack of prioritization and monitoring of medication safety and a poor pharmacovigilance system contributes towards the under-reporting of ADRs, the Pakistani pharmacovigilance system is not an exception [14]. Pakistan became a full member of WHO’s Program for International Drug Monitoring (PIDM) in 2018, but the provision of medicines’ safety and ADR reporting remains a challenge [15]. 

Qualitative research is aimed at the exploration and understanding of people’s experiences of a certain phenomenon through the use of non-numerical data [16]. Although the field of clinical and healthcare research is dominated by quantitative research, quantitative methods alone are not sufficient [17]. Thus, to get a better understanding of the issues, it is important to understand and be able to conduct qualitative research. This research does not traditionally include numbers and statistical figures, or “count” data [18].

According to the best of our knowledge, this is the first-ever qualitative study to evaluate physicians’ knowledge, practices, and possible barriers that hinder the process of adverse drug reaction reporting in Lahore, Pakistan.

## 2. Materials and Methods

### 2.1. Ethical Approval

The study was approved under reference no. HEC/PUCP/1943 by the Humans Ethics Committee (HEC), University College of Pharmacy, University of the Punjab, Lahore, Pakistan.

### 2.2. Study Design

We adopted a qualitative research approach, since a qualitative analysis provides an in-depth understanding of the interaction between the individuals and their experiences [19]. It focuses on the data that is non-numeric, allowing the researcher to get an in-depth insight into the respondent’s viewpoint [20]. Additionally, qualitative analysis aids in the identification of the gaps that remain unnoticed by the survey-based research methods [21]. 

### 2.3. Study Sampling and Sampling

The targeted participants were recruited from public hospitals in Lahore, including four major tertiary care public hospitals, and relevant approval was obtained from the heads of the department. The study was carried out in tertiary care hospitals in Lahore, the capital city of Punjab Province of Pakistan. Participants who were registered physicians from Pakistan Medical and Dental Council and working as full-time permanent employees in a tertiary care hospital were recruited for the study. Participants in our study were purposively selected based on their convenience as per time and place availability with prior experience and an understanding of the health systems [22,23]. During recruitment, the selected participants were aware that participation for the interview was voluntary, and they were free to decline the participation. Besides, no incentive for participation was offered to the participants.

### 2.4. Data Collection

A semi-structured interview guide was developed based on the review of the literature [8,10,12,13,24,25,26,27]. It was tested for its validity and reliability by two experienced researchers at Universiti Sains Malaysia, Penang, Malaysia. It was pre-tested and modified after the pilot results (Appendix A). Table 1 presents a summary of the topic guides for the semi-structured interview.

An explanatory statement, detailing the study objectives, was given to each physician, and written consent was taken from them. The personal information of the participants was taken on a separate data collection form. The principal author of the study interviewed the participants at their workplace between February 2018 and May 2018. The interviews were conducted in the English language, while each interview lasted for 30–40 mins. Appropriate probing questions were asked where necessary to seek more information. All interviews were audio-recorded and transcribed verbatim. Data saturation was achieved after 11 interviews; however, two additional interviews were conducted to see if new themes were emerging [28]. 

### 2.5. Analysis

The data were thematically analyzed according to the method described by Braun and Clarke [29]. The data were analyzed manually by the reading and re-reading of the interviews, and an inductive and flexible approach was undertaken by the research team. The assigned co-authors read interview transcripts to confirm that the generated codes and themes were truly reflective of the content of the interviews, and a mutual consensus was reached among all assigned research team members. A summary of the different phases of analysis is presented in Table 2.

### 2.6. Reporting

The COREQ (consolidated criteria for reporting qualitative research) checklist for reporting qualitative studies aided the reporting of the methods and results [30].

## 3. Results

### 3.1. Demographic Details of the Participants

**Figure 1:**
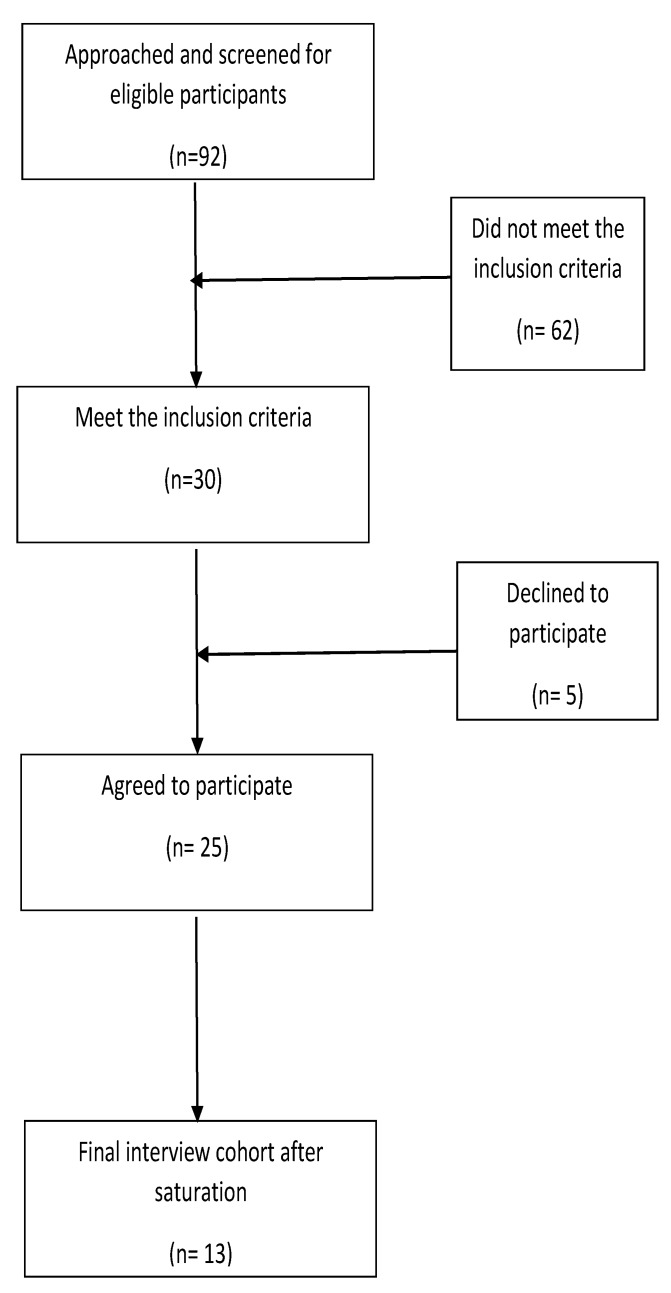
A flow diagram of the participants’ recruitment for the qualitative interviews.

A total of 13 physicians aged between 25 and 55 years were interviewed. A flow diagram of the participants’ recruitment is given in Figure 1.

There were 5 female participants and 8 male participants. The majority of the participants (*n* = 10) were more than 30 years of age, while the rest belonged to the age group below 30. Four participants had an experience of 1–5 years, while five physicians had an experience of 10 or more years of service. Nine of the participants had either completed or were completing a specialization in their field as Fellows of College of Physicians and Surgeons I and II, while four participants were graduates with a master’s degrees in public health. The majority of the participants had reported ADR in their work setting (either in verbal or in written form). The demographic distribution of the participants is described below (Table 3).

### 3.2. Thematic Analysis of the Content

The thematic content analysis of the interview resulted in six major themes. These themes are listed as: (1) Familiarity with medication safety & ADR concept, (2) Knowledge about pharmacovigilance activities, (3) Practices related to ADR reporting, (4) Barriers to ADR reporting, (5) Acknowledgement of the pharmacist’s role, and (6) System change needs.

#### 3.2.1. Theme 1: Familiarity with Medication Safety and ADR Concept

##### Subtheme 1: Knowledge about the Definition

The participants were interviewed regarding their knowledge and perceptions about medication safety, and here are the responses:

*“Medication safety is the use of a drug causing no harm to the patients.”*

(Phy-7)

*“It means if a drug is given to the patient, there should not be any harm, I give medicines to treat the patient. For me, safety is more important than the drug.”*

(Phy-10)

When the participants were asked to define an adverse drug reaction, they stated:

*“It is any reaction of the body to the drug which is harmful to the body that is what I can simply define.”*

(Phy-6)

*“An ADR is a reaction which is unwanted after taking the medication.”*

(Phy-11)

##### Subtheme 2: Perceptions towards Types of ADR Need to be Reported

Participating physicians gave mixed responses about the types of ADRs they report, as some were of the view that only major and life threatening reactions should be reported, while others said that every ADR should be reported:

*“Drug allergies or drug causing arrhythmias, cardiopulmonary arrest or any drug causing life threatening conditions, it should be reported. For minor reactions, we don’t bother.*

(Phy-5)

*I think every sort of ADR should be reported either its minor or major.”*

(Phy-7)

#### 3.2.2. Theme 2: Knowledge about Pharmacovigilance Activities

##### Subtheme 1: Knowledge about ADR Reporting

The majority of the physicians had good knowledge about ADR reporting and its importance. However, many of them expressed that they did not report any ADR and described their views as:

*“We have guidance and basic knowledge about ADR reporting. There is a form, it’s available in pharmacy, in case of any ADR, we fill the form and send it through the proper channel to AMS (Assistant Medical Superintendent) and then it is sent back to pharmacy for the medicine evaluation.”*

(Phy-5)

*“No guidance, as there is no protocol, there is no proper reporting system in tertiary care hospital of Lahore.”*

(Phy-7)

##### Subtheme 2: Knowledge about ADR Reporting Center

Regarding their knowledge about the presence of ADR reporting centers in Pakistan, the majority of the physicians were unaware of the national pharmacovigilance center, while a few acknowledged the presence yet had very little information about it:

*“No, I am not aware about this body.”*

(Phy-3)

*“I am not aware because I never heard about any system of adverse drug reaction reporting maybe the system is not in practice, I haven’t seen anyone around me reporting.”*

(Phy-6)

*“Yes, I am aware about the system, but I am not in touch. I really don’t know the name but there is some drug regulatory authority.”*

(Phy-10)

#### 3.2.3. Theme 3: Practices Related to ADR Reporting

When the physicians were asked to speak about their practices regarding adverse drug reaction reporting in the hospital setting, they replied as follows:

*“We usually inform the pharma companies and also we discuss with the other colleagues as well, but we do not report in a particular documented way.”*

(Phy-3)

*“We verbally report to each other but do not document this as such.”*

(Phy-6)

*“Yes, we note it down but do not report.”*

(Phy-8)

#### 3.2.4. Theme 4: Barriers to ADR Reporting

Work overload, lack of communication, lack of knowledge and training, and fear of legal liability were identified as the major pitfalls of the current healthcare system. 

##### Subtheme 1: Impact of Workload

The majority of the physicians identified an increased workload as a major barrier for adverse drug reaction reporting:

*“Our health system is a barrier, and workload are another barrier. Basically these 2 barriers will fit everywhere in the system.”*

(Phy-4)

*“Time is one of the factors, if I am sitting in outdoor patient department, I have to see 100 patients in a day and have 4 hours to see them then what will you expect from a person, so they cannot report.”*

(Phy-7)

##### Subtheme 2: Lack of a Reporting System

All interviewed physicians identified the lack of a reporting system as a major barrier that causing hindrance in the smooth reporting of ADRs. They expressed their views as:

*“First of all, no proper system is there to whom we can report. There is nothing which is bounding or stopping me from reporting it’s just that there is no so system that is why we do not report.”*

(Phy-6)

*“I think lack of system, as we don’t have any reporting system and we are not taught to do it.*

(Phy-10)

##### Sub-theme 3: Lack of Education

Many physicians admitted that the cause of not reporting an ADR can be linked up with the lack of education and training. It was very interesting that especially the physicians who were in their early 30s admitted to this fact:

*“Barriers are not there, personally speaking about my opinion. Merely ignorance is there. We are not being educated at this level. Being a physician or a surgeon, we are conducting our jobs. But we are not educated properly that when, how and where to report?”*

(Phy-3)

*“The system in which I have studied and worked, I have no idea about ADR reporting and even about the yellow card. It is a lack of education and my training.”*

(Phy-4)

##### Sub-theme 4: Legal Liability

The majority of the physicians identified a fear of legal liability as a contributing factor towards less or no reporting on ADRs:

*“Legal liability is another barrier, nowadays people are aware of all these things and they know what is happening and they seek the legal actions also against the companies and sometime against the practitioners.”*

(Phy-1)

*“We do not report, because in a public hospital if I report anything, it is going to be a risk at my professional carrier. We verbally report to each other but do not document this as such.”*

(Phy-5)

##### Sub-theme 5: Peer Pressure

Many of the physicians stated that ADR should not be reported without the involvement of a senior colleague. They expressed that this was a hindrance as seniors were not present when the ADR happened, and, hence, they cannot decide whether they should report it or otherwise:

*“No, we have not been told about the protocol because, if some kind of drug reaction had occurred and if we face any kind of difficult thing, we just have to report our seniors. We have not been told about reporting.”*

(Phy-9)

#### 3.2.5. Theme 5: Acknowledgement of Pharmacist’s Role

Participants were asked about the lead role for medication safety issues; many physicians were of the view that a pharmacist should be in charge of medicine safety issues in the wards, as they are more educated and trained on that particular issue:

*“Clinical pharmacist is needed in the wards because he can really take care of the event because they have studied in detail about the pharmaceutical aspects of drugs, but we did not.”*

(Phy-5)

*“Every hospital should have pharmacist to tell the staff about the safety of the drug and the brand of the drug. If some adverse effects occur pharmacist should be knowing those things and should be a bridge between the company and physician, the safety of the drug will be improved.”*

(Phy-10)

#### 3.2.6. Theme 6: System Change Needs

Physicians shared their views about change in the ADR reporting system via an improvement in training, and they recommended to establish an online reporting system in the country:

*“Like if the government has introduced CME (Continuing Medical Education) concept for doctors, the same way they should have some drug reactions like learning thing as a condition for the renewal of the registration. It would be a great step in improving the adverse reaction reporting by doctors.”*

(Phy-4)

*“Well, need is there to develop a system where we can report ADRs and can get the feedback directly and promptly. The system should be linked throughout the country for the maximum coverage. Hence, this will make us to come up with some solid data on adverse drug reaction at national level.”*

(Phy-3)

## 4. Discussion

The present study has identified several issues in Pakistan’s pharmacovigilance system. The results of the study have displayed that participants had an idea about medication safety and adverse drug reaction reporting. This is very similar to a study in northern Sweden, by Backstrom in 2000, which showed that the majority physicians had a good knowledge about the ADR reporting system [31]. However, our study reports that the interviewed physicians were not aware of the local pharmacovigilance centers in the country, nor of the activities performed by these centers [32]. These findings are consistent with a study in Malaysia, which found the predictors of under-reporting of ADRs by physicians. According to which, about 40% of the respondents were completely unaware of the national pharmacovigilance centers; hence, they did not report an ADR [33]. This shows the lack of communication between the administrative bodies of the centers and the hospital staff. One of the measures to address this is to introduce pharmacovigilance as an essential part of the training of healthcare professionals, especially among physicians. Furthermore, national pharmacovigilance centers should publicize their activities among physicians, as has been mentioned in the literature [34]. The Uppsala Monitoring Center in Sweden has provided online educational support, both for students and healthcare professionals, in terms of web-based lectures as well as seminars on signal detection and causality assessment [35]. These resources could be used to strengthen the knowledge of physicians and other healthcare professionals regarding the reporting of an ADR and to improve a pharmacovigilance system in the country [36].

It was found in our study that all interviewed physicians had a similar behavior toward reporting an ADR in their practice setting. They usually report an adverse drug reaction on the patient’s information sheet. Sometimes, they discuss the problem with the pharmaceutical companies or may pass this information to the hospital administration or medicine purchasing staff; however, it was observed that they did not fill up an ADR form. This also showed that there was absolutely no involvement of a pharmacist in reporting or discussing the ADR events. Physicians were mostly colluding with the drug companies to share information about adverse drug reactions if anything happened. The problem can be ruled out by the implementation of the pharmacist’s role in the wards and less involvement of pharmaceutical companies with the physicians. Pharmacovigilance revolves around the safety of medicines, and pharmacists play a crucial role in ensuring the rational and safe use of medicines. Hence, an effective use of pharmacist’s role will improve pharmacotherapy outcomes [37].

During the interviews, participants identified many barriers to ADR reporting in the hospital setting. The most discussed barrier was the increased workload on physicians. This has also been identified in other studies, and the workload is regarded as one of the obstacles to the spontaneous reporting of ADRs by healthcare professionals [31,38]. Lahore, being the central hub of Punjab, caters to the maximum number of patients from all around Punjab. This puts extra pressure on tertiary public hospital working staff including physicians. According to a survey report, the doctor-patient ratio in Punjab is 1:1702, which is too far to meet the standard provided by the WHO’s standard of a 1:1000 physician-patient ratio [35]. The statistics given in the report do not show the actual number of physicians working in the field; rather, they are just reflective of physicians who are registered with the Pakistan Medical and Dental Association [39]. Hence, an increase in the number of physicians in the country, especially in tertiary care hospitals, would help solve the problem.

Another key barrier identified in our study was the lack of knowledge about the importance of pharmacovigilance. This could be associated with the undergraduate training in pharmacovigilance. It was observed that medicine risk perceptions are either insufficient or improperly delivered to prepare the physicians for the ADR monitoring and reporting in their future career, as has been seen in other studies [7,38]. Ineffective communication between physicians and administrative healthcare authorities was also identified as one of the reasons for not reporting [34]. These barriers can be overcome by including pharmacovigilance teaching as a mandatory part of the curriculum and by providing training and information about pharmacovigilance to physicians. 

An absence or lack of a reporting system was found to be another contributor impeding the smooth reporting of ADRs. The participants felt that they lack an appropriate system regarding the reporting of an ADR, as identified in various studies [40,41,42,43]. A study by Stoynova in 2013 among Bulgarian physicians identified a lack of knowledge about the ADR reporting system, and this was classed as the main barrier for not reporting an ADR [8]. The problem can be resolved by implementing an effective online system for the reporting of ADRs in every hospital. This can then be efficiently disseminated into the national level reporting.

In our study, we found that the majority of physicians had a fear of legal liability in case of reporting of an ADR and may have considered reporting as a downfall to one’s career. In 2015, Aljadhey highlighted the same issue in a study among Saudi physicians, pharmacists, and academicians, whereby the participants identified workplace culture as a barrier to ADR reporting [38]. Hospital management and the relevant healthcare authorities can play an important role in overcoming this barrier if they take appropriate actions and safeguard the anonymity of the reporter of an ADR. Flemons & McRae in 2012, described that the hospital management should develop a culture of ADR reporting by establishing a well-organized and well-managed reporting system [44]. In Pakistan, the Drug Regulatory Authority of Pakistan (DRAP) has introduced an online reporting form for both healthcare providers and patients. However, the form needs some modification regarding the explanation of the privacy of the healthcare professionals who are reporting an ADR [45].

An interesting barrier towards ADR reporting was found to be the involvement of the senior physicians in ADR reporting. Physicians are trained by their senior colleagues, and they usually rely on them for decisions and consider this as social support. This helps in getting better training from the seniors, but sometimes also imposes pressure from seniors [46]. Hence, there is a need to provide a system that socially supports but at the same time empowers physicians (especially juniors) to perform their duties without hindrance or any pressure from the senior doctors.

The findings from the study suggested a positive attitude of physicians towards reporting an ADR, which is very positive indeed, as was observed in other studies, where participants were willing to learn and apply ADR reporting knowledge in their work setting [8,11]. This can be related to the theory of planned behavior, where the behavior of the individual is contributed by three main predictors, including the attitude, subjective norm, and perceived control of behavior [47]. These three contributors create an intent to perform a certain behavior. The theory explains that a positive attitude, favorable social norm, and high level of perceived behavioral control are the best predictors for the intentions to follow a certain behavior. The likelihood of a certain behavior decreases if any of the three predictors are unfavorable [48,49]. Thus, intentions of physicians to report an ADR are greatly influenced by the physicians’ attitudes, subjective norms set by their senior or colleagues, and the ability to exhibit a certain behavior toward the reporting of an ADR.

It was interesting to know that the physicians now understand the expanding role of the pharmacist and that they would like them to be a part of the healthcare team. In the past, the pharmacist has been the most underutilized healthcare professional; however, this is now changing, as pharmacists claim more space in the healthcare milieu [38,50,51]. This is in line with a study where both nurses and doctors referred to the pharmacist to provide advice regarding drug management and ADR reporting [52].

The respondents in our study suggested reforms for the improvement of the ADR reporting system in the country, including continuous education, seminars, and the delivery of training courses, as continuing education is an important tool for increasing physicians’ awareness of ADRs. Oshikoya and Awobusuyi in 2009 also recommended including pharmacovigilance as a topic in continuing education programmes [7]. Various studies have indicated that the optimization of the knowledge, attitude, and practices about pharmacovigilance is essential to promote reporting [53,54,55,56]. Furthermore, the participants also expressed having an online system of ADR reporting in each healthcare center. This could connect and disseminate the ADR reports throughout the country by following standard operating procedures. Respondents suggested reforms for the improvement of the ADR reporting system, including continuous education, seminars, as well as training courses. The literature also supports that a provision of optimal knowledge, awareness of attitude, and practices related to pharmacovigilance are essential to promote ADR reporting [53,55,56]. The participants also urged the government to take vital steps to ensure a safe and effective medicine utilization among the population [57].

Globally, there is a shift of ADR reporting from the prescriber to the consumer or patient in developed countries. However, in developing countries like Pakistan, the ADR reporting system is still at its infancy stage [58,59]. Thus, the current study is meant to highlight the issues responsible for the weak ADR reporting system. We believe that the outcome of this study would further help on two levels: (a) it will strengthen the current role of healthcare professionals in ADR reporting, and (b) it will also provide a guideline and would shift the onus of ADR reporting from the prescriber to the consumer in the future.

### Strengths and Limitations

This study has provided a useful and detailed insight into the knowledge, attitude, and practices of physicians towards pharmacovigilance activities in Lahore, Pakistan. A key strength of this study was the inclusion of physicians from four tertiary care settings. This facilitated an in-depth investigation of the range of challenges encountered in most facility-based healthcare settings. Particularly novel was the consideration of physicians as participants for the study, as no previous qualitative study has been done on this particular issue in Pakistan. Therefore, the current study has addressed this significant gap in the literature. The sampling strategy ensured that a full range of experiences and perspectives were elucidated. Besides, the data analysis based on a thematic analysis approach facilitated the generation of rich, detailed, and firmly grounded findings. Hence, it was found most appropriate given the limited literature availability and the complete absence of qualitative data on the topic within the Pakistani setting. Besides, there are a number of potential limitations associated with this study. It is possible that the interviewer was a pharmacist, which may have resulted in socially desirable responses from the participants. However, no relationship was established with the participants prior to the interview, and participants only had knowledge of the interviewer in the capacity of a researcher; this would have helped to overcome the potential limitation of a response bias. Participants were assured that their anonymity and confidentiality would be maintained, and it was evident during the interviews that participants expressed their opinions freely and honestly. Another limitation in our study was that it was carried out in Lahore; therefore, the results of the present study cannot be generalized to represent the other provinces in the country. However, Punjab is the most developed province, and Lahore is the second biggest and most developed city in the country. Thus, it is expected that the results in the other parts of the country would not be very different or may be worse.

## 5. Conclusions

The outcome of this study has showed that physicians know about medication safety; however, the reporting of adverse drug reactions is low and constitutes a challenge. The major barriers to reporting include a lack of knowledge of ADR reporting, a lack of training, the work environment, and the workload. Physicians showed a willingness to report ADR provided the hospitals have an updated and uniform reporting system in place under the supervision of pharmacists. The findings of this study are important, and they can help in the development of better policies to promote a better pharmacovigilance system in the country.

## Figures and Tables

**Table 1:** Summary of the topic guide for the semi-structured interview.

No.	Summary of the Interview Topic Guides
1	Knowledge and perceptions about Medication safety
2	Knowledge, attitudes and practices about Adverse Drug Reaction (ADR) reporting
3	Knowledge about Adverse Drug Reaction reporting system
4	Future directions

**Table 2:** Data analysis process.

Phase of Analysis	Tasks Completed	Research Team Member Involved
Phase 1: Data familiarization	Transcription, reading and re-reading of interview transcripts.	RH
Phase 2: Initial codes generation	Initial, open coding of entire data set	RH and MAH
Phase 3: Search for themes	Categorization of codes into potential themes	RH and MAH
Phase 4: Review of themes	Confirming themes—ensuring the internal homogeneity and external heterogeneity of themes.	RH, discussed with MAH and FH.
Phase 5: Defining and naming themes	Further refinement of themes	RH, confirmed with MAH and FH
Phase 6: Report finalization	Production of the manuscript, selection of illustrative quotes	RH, reviewed by and discussed with MAH.

**Table 3:** Demographics of the participants.

Characteristics	Frequency
Gender	
Male	8
Females	5
Age (Years)	
20–30	3
31–40	5
>41	5
Education	
Graduation	4
Specialization	9
Experience (Years)	
1–5	4
5–10	4
>10	5
ADR reporting	
Yes	8
No	5

## Appendix A

Physicians’ interview guide

Objective: Exploring the knowledge & perceptions of physicians towards medication safety & Adverse Drug Reaction (ADR) reporting

### Part I

Focus: Knowledge and perceptions about Medication safety

1. What comes into your mind, when you hear the word “Medication Safety”.
2. Do you counsel patients on medication safety? If yes which particular aspect of it, under what conditions do you counsel them and why? (condition mean disease state)
3. Any recent counselling being offered to patients, any examples (prompt for conversation, symptoms, recommendations/advice given)

### Part II

Focus: Knowledge and attitudes about Adverse Drug Reaction (ADR) reporting

1. Do you know, what is Adverse Drug Reaction? How would you define it?
2. What would you do if you were approached by a patient with a severe ADR (any recent incidence, what was your strategy to deal with the patient)?
3. What type of adverse drug reactions you consider should be reported?
4. In your current practice, how many ADR cases you have seen? (Did you report/ record them by yourself or heard it from some other colleague)
5. In your organization, who is responsible for ADR reporting?
6. Do you have any guidance on reporting or how to and when to report any ADR? And report to whom and Where?
7. Have you ever sent an adverse drug reaction report to your national reporting agency, when that happened and what is the reason behind?
8. Have you ever sent an adverse drug reaction report to the responsible pharmaceutical company, when that happened and why did you send it?
9. What are the factors that you think can impact and may encourage physician to report ADRs (why a physician should report an ADR)?
10. In your opinion, what are the possible factors that contribute as the barriers to ADR reporting?
11. Do you think your job in any way makes it easy/difficult to report ADR.
12. What type of medicine information resources (journal, news, formulary{BNF}) you prefer while reporting ADR?
13. Do you receive and routinely review publications to become aware of medications with error potential?

### Part III

Focus: Knowledge about Adverse Drug Reaction reporting system

1. Are you aware about the existence of the regulatory body that regulates ADR reporting in Pakistan? Reasons? if you are not aware? (education/training)
2. In your opinion, do you think that there is a need to change the system about medicine safety and ADR reporting? What benefit would it have?

### Part IV

Focus: Future perspective

1. Do you think that your role has changed and how it can contribute towards medication safety?
2. What could be the possible suggestions to improve ADR reporting in hospital setting in future?

### Conclusion/Suggestions

Would you like to provide any additional comments about medication safety and ADR reporting system in Pakistan?
